# Development of a reliable and construct valid measure of nutritional literacy in adults

**DOI:** 10.1186/1475-2891-6-5

**Published:** 2007-02-14

**Authors:** James J Diamond

**Affiliations:** 1 Department of Family and Community Medicine, Jefferson Medical College, 1015 Walnut Street, Suite 401, Philadelphia PA 19107, USA

## Abstract

**Background:**

Research into the relation of literacy to health status has not included measures of nutritional literacy. This may be a critical area in the study of chronic conditions such as hypertension and diabetes, which can both relate to obesity and nutrition. This paper details the development and psychometric characteristics of the Nutritional Literacy Scale (NLS), offered as a measure of adults' ability to comprehend nutritional information.

**Methods:**

In order to assess the internal consistency and construct validity of the NLS, demographic data, readability statistics, NLS scores and scores on the Reading Comprehension Section of the Short Test of Functional Health Literacy in Adults (S-TOFHLA) were collected in a cross-sectional study of 341 patients from two primary care practices.

**Results:**

The NLS score showed acceptable internal consistency of 0.84 by Cronbach's alpha coefficient. The Pearson correlation between the NLS and the S-TOFHLA was 0.61, supporting evidence for construct validity.

**Conclusion:**

Given the importance of proper weight and nutrition in the health of the public, as well as the absence of research on literacy skills as related to nutritional concepts, the NLS has the potential to add to the national research agenda in these areas.

## Background

While there has been a decrease in mortality from cardiovascular diseases in the United States dating back at least 40 years, there have been increases in the prevalence of diabetes and obesity, both of which pose significant cardiovascular risks [1]. The CDC estimates the prevalence of diabetes (diagnosed plus undiagnosed) in 2002 at more than 8% of the population older than 19 and more than double that for people over 64 [2]. That obesity is increasing is also well-documented, and is now estimated at approximately one-third of the US population ages 20 to 74 [3].

The increased prevalence of obesity and Type 2 diabetes has been found to relate to poor dietary patterns and inadequate exercise. The Diabetes Prevention Program (DPP) has clearly shown that a lifestyle intervention centering on diet and exercise can reduce the likelihood of the onset of Type 2 diabetes [4].

Given the rising prevalence of conditions such as diabetes and obesity, plus the role of nutrition and diet in their etiology, understanding whether the ability to comprehend nutritional information contributes to health status is highly significant. Furthermore, better understanding of the factors leading to poor dietary and lifestyle patterns is critical so that effective educational and interventional programs can be developed.

In parallel with the epidemiology of obesity and diabetes, interest has developed around the topics of health literacy, health policy, and medical outcomes [5]. In 2003 Parker, Ratzen, & Lurie described the policy challenges relating to health literacy, as well as strategies for dealing with the problem of inadequate literacy, especially among those in greatest need [6]. Because health literacy research is a relatively new field, there have been several definitions of *health literacy *and ongoing discussions about the construct and its measurement [7]. And there would be agreement among researchers that no single instrument that will measure all aspects of the construct. Nonetheless, the "crucial link between literacy and health" has been well-documented by Wilson [8]. As but one example, among a great many, Schillinger and colleagues demonstrated that adequate health literacy was associated with achieving tight glycemic control (HbA1c below 7.3%) [9].

According to Baker's recent conceptual model, the work described in this paper would be labeled as health-related print literacy [7]. Other investigators have focused on the ability of adults to understand nutrition labels [10,11]. This is important as it helps us to understand literacy outside the textual domain since labels can be both graphical and numerical. Not all investigators have related findings regarding labels to medical outcomes. In addition, consumers and patients receive nutritional information from multiple, non-label sources, including magazines, newspapers and the Internet.

This paper describes the development of a reliable and valid scale to measure a construct being named nutritional literacy. Given the rising prevalence of nutritionally-related conditions such as obesity and diabetes, such a scale has the potential to contribute to the study of the relation of literacy to health status.

## Research methods and procedures

The Reading Comprehension Section of Short Test of Functional Health Literacy in Adults (S-TOFHLA), one of the commonly-used and validated measures of health literacy, was used as a model for the Nutritional Literacy Scale (NLS) [12]. The first version of the NLS was constructed from declarative sentences found in several nutritionally-related websites such as Mayo Clinic's Food and Nutrition Center, Tufts Nutrition Navigator and the USDA Center for Nutrition Policy and Promotion. Because the initial version of the NLS was used in a project on the metabolic syndrome, the items reflected cardiovascular-related topics such as "heart-healthy" eating, saturated fats, and portion size. Following the model of the S-TOFHLA, the modified Cloze procedure was used in which one or more words are removed from a sentence. Each sentence then includes several different options and the respondent picks the one that "fits" the best. For example, a sample item such as "Losing __________ can be a challenge" might be followed with choices such as (A) weight, (B) calories, (C) fiber, and (D) vitamins listed in a multiple-choice format. The Cloze procedure has been used for many years as one way to measure reading comprehension [13].

The initial version of the NLS contained 21 modified-Cloze items in four-option multiple choice format and was pilot-tested on 132 adults, including family medicine patients, people taking courses at a local university, municipal employees and community members. The pilot subjects were able to complete the NLS without difficulty. However, a few of the items needed to be revised so as to better reflect comprehension, rather than knowledge of nutritional facts. A revised NLS containing 22 items was used in a second study with 103 adult patients in a family medicine practice different from the pilot site. To aid in the assessment of construct validity, the patients also completed the S-TOFHLA. Their medical charts were abstracted for information on cardiovascular variables such as the diagnoses of hypertension or diabetes. As evidence of construct validity, the two literacy measures – health and nutritional – were correlated (r = 0.69). Also, the patients with diabetes or hypertension had lower literacy scores than those without these diagnoses. The NLS also showed reasonable internal consistency by Cronbach's alpha coefficient (0.83).

Based on these data, a review of the item responses and to increase content validity, the scale was lengthened to 32 items. The scale was subsequently shortened to 28 items, following suggestions from a journal reviewer and an assessment of the statistical data for each item. Content areas such as organic foods, fiber, calcium and sugar were added to the original version. In general, items within each content area are ordered from the easiest to the more difficult, based on the pilot data. A total, number-right score is used for analysis. A page of demographic questions on age, gender, ethnicity and education is included. Because one goal in developing the scale was to be able to have patients complete it in the clinical office or by mail, the scale is untimed.

The 28-item version of the NLS was completed between 2004 and 2006 by 341 patients in four separate administrations. Three groups of patients were part of a University-based family medicine practice. One of these groups (Group 4) specifically included overweight and obese patients. Group 2 consisted of patients in an integrative medicine practice. They were included because they were known to be interested in nutritional issues, and because they were drawn from a less diverse population than were the family medicine patients. Because the groups were demographically diverse, combining them yielded a dataset that was potentially "richer" for analysis purposes. For three of the groups, patients were approached in the office by an assistant who requested their participation. While there were no explicit *exclusion *criteria for these groups, the assistants work in the office on numerous studies and are able to exclude patients in distress or those who are thought to be unable to complete the tasks based on their responses to the verbal consent script the assistants use. Although a random selection protocol in the office is impractical, the overall demographics of the practice were known and during the data collection phase, groups of patients such as younger men were targeted to try and keep the overall group representative. Patients in three of the groups also completed the Reading Comprehension Section of the S-TOFHLA, and they were given a phone card as an incentive. Because the providers in Group 2 did not want to place too much of a burden on their patients, they did not complete the S-TOFHLA. Lastly, the patients in Group 3 completed the scales by mail. Because of the potential introduction of "method variance," these data were originally not going to be included. However, as there was substantial overlap in the distribution of scores for this group and the others, they were included in the final analysis.

The item responses were entered into an Excel workbook and checked for accuracy. For the analysis, all the worksheets were imported into a statistical package (SAS Version 9.1 for PCs). The Pearson correlation between the two literacy measures was calculated as the estimate of construct validity of the NLS, with Cronbach's alpha as the estimate of internal-consistency. All data collection and consent procedures were approved by the University's Institutional Review Board.

## Results

Reflecting the practices' demographics, 78% of the respondents were female. Seventy-one percent had more than a high school education. The overall mean NLS score was 23.7, with a median of 25.0 and a standard deviation of 4.1. See Table 1. Although we did not hypothesize any group differences, we expected that Group 2 might score higher than the others, as this was the most highly educated and considered to be the most interested in nutrition. Group 4 included overweight and obese patients, and with the least education, they might have been expected to score lower that the others. In order to look more closely at the differences in NLS scores among the four groups, we ran a one-way ANOVA, with Group as the independent variable. The overall F of 6.74 had a p-value of 0.0002. The Tukey HSD test showed some interesting results. Groups 1 and 3 were the most typical groups of family medicine patients. Their mean literacy scores did not differ, lending support for combining these groups even though their data were collected using different methods. Generally speaking, the group of overweight and obese patients did score lower than the others, and the group of patients known to be interested in nutrition did score higher than the others. While these results need to be replicated, they do lend support to the validity of inferences that can be made from the NLS. This might be especially supportive of NLS validity, since the S-TOFHLA scores (available for three of the four groups) did not follow the same pattern. For example, Group 4 (overweight or obese patients) did not have the lowest S-TOFHLA mean score, even though their mean NLS score was the lowest. Lastly, the NLS mean scores did not differ by gender or age, but did correlate with self-reported years of education (0.41 by Pearson's product-moment correlation).

**Table 1:** Patient Demographics and Nutritional Literacy Descriptive Statistics

	GROUP 1 (n = 72)	GROUP 2 (n = 52)	GROUP 3 (n = 97)	GROUP 4 (n = 120)	TOTAL (n = 341)
MEAN (SD) NLS	24.5 (3.0)	25.5 (2.4)	23.2 (4.5)	22.8 (4.5)	23.7 (4.1)
MEDIAN NLS	25	26	24	24	25
MEAN (SD) AGE	58.8 (11.7)	51.0 (14.1)	41.7 (15.4)	40.3 (13.5)	46.4 15.6)
MALE %	19	8	26	26	22
CAUCSN %	39	86	34	20	38
EDUC % >HS	64	90	75	64	71
RELIABILITY*	0.72	0.69	0.86	0.85	0.84
VALIDITY**	0.65	--	0.59	0.69	0.61

* Cronbach's coefficient alpha

** Pearson product-moment correlation between NLS and S-TOFHLA scores. Group 2 did not complete the S-TOFHLA.

Supporting the internal consistency of the NLS, the overall combined-groups Cronbach's alpha coefficient was 0.84. As one standard for construct validity, the Pearson correlation between the NLS and the S-TOFHLA scores was 0.61 for all groups combined. According to the standards for the S-TOFHLA, the overall group was quite literate. Only 2% scored in the inadequate range; 5% in the marginal and 93% in the adequate.

## Discussion

The NLS has acceptable psychometric characteristics. From a content validity perspective, the NLS covers the major consumer-related topics in nutrition. The NLS correlates with the S-TOFHLA Reading Comprehension score, providing evidence of construct validity. Given that the NLS is intended as a research tool rather than for individual diagnosis, the internal-consistency estimate of 0.84 is excellent [14].

The readability statistics for the NLS are also appropriate for an adult English-speaking population. Per Microsoft Word, the reading ease score of the NLS was 69.7 which is well within the range recommended. The grade level score of 6.7 indicates that the patients in this study, 71% of whom had at least a high school diploma, had little difficulty reading the items.

Some limitations of the NLS exist. For one, there was no pre-assessment of the decoding skills of the patients. It is possible that some may not have been able to read. While participation was voluntary, non-readers might have been included inadvertently. We believe this was not a large problem, as there was only one score below 7, which is the expected score from random guessing of all 28 four-option items. Also, very few patients refused to participate. Those that did might have felt they had poor reading skills or difficulty seeing and self-selected themselves out of the study. The scale is only for adults and has not been adapted for children who might require a format other than traditional paper and pencil. We do plan to pilot the NLS with high school and college students, and to collect data using a Spanish version that is now available. We are currently working to document the empirical validity of the inferences made from the NLS. In our pilot data, the patients with chronic conditions such as diabetes and hypertension did have lower NLS scores that the patients without these diagnoses. Since both of these conditions can be related to obesity, such a relationship would be expected. Future research with the NLS will analyze measures of health beliefs, dietary intake, and a chart review.

## Conclusion

The current version of the NLS reflects both reliability and judgmental validity standards that demonstrate it can potentially be used in research on literacy and health status among adults. Future research will determine the "value added" by this new instrument to our understanding of the contribution of nutritional literacy to the measurement of health status and outcomes. Given the importance of proper weight and nutrition in the health of the public, as well as the absence of research on literacy of nutritional information, the NLS has potential to add to the national research agenda in these areas.

## Competing interests

The author(s) declare that they have no competing interests.

## Authors' contributions

JJD is responsible for the conception and design of the Nutritional Literacy Scale. He supervised the assistants collecting the data and did all the data entry, analysis and preparation of the manuscript.
